# The emerging role of KDM5A in human cancer

**DOI:** 10.1186/s13045-021-01041-1

**Published:** 2021-02-17

**Authors:** Guan-Jun Yang, Ming-Hui Zhu, Xin-Jiang Lu, Yan-Jun Liu, Jian-Fei Lu, Chung-Hang Leung, Dik-Lung Ma, Jiong Chen

**Affiliations:** 1 State Key Laboratory for Managing Biotic and Chemical Threats to the Quality and Safety of Agro-Products, Ningbo University, Ningbo, 315211 Zhejiang People’s Republic of China; 2 Laboratory of Biochemistry and Molecular Biology, School of Marine Sciences, Ningbo University, Ningbo, 315211 People’s Republic of China; 3 Key Laboratory of Applied Marine Biotechnology of Ministry of Education, Ningbo University, Ningbo, 315211 People’s Republic of China; 4 Institute of Chinese Medical Sciences, State Key Laboratory of Quality Research in Chinese Medicine, University of Macau, Macao SAR, People’s Republic of China; 5 Department of Immunology and Medical Microbiology, Nanjing University of Chinese Medicine, Nanjing, 210046 People’s Republic of China; 6 Department of Chemistry, Hong Kong Baptist University, Kowloon, Hong Kong, 999077 People’s Republic of China

**Keywords:** KDM5A, Cancer, Jumonji C domain, Histone methylation, Drug resistance, Targeted therapy

## Abstract

Histone methylation is a key posttranslational modification of chromatin, and its dysregulation affects a wide array of nuclear activities including the maintenance of genome integrity, transcriptional regulation, and epigenetic inheritance. Variations in the pattern of histone methylation influence both physiological and pathological events. Lysine-specific demethylase 5A (KDM5A, also known as JARID1A or RBP2) is a KDM5 Jumonji histone demethylase subfamily member that erases di- and tri-methyl groups from lysine 4 of histone H3. Emerging studies indicate that KDM5A is responsible for driving multiple human diseases, particularly cancers. In this review, we summarize the roles of KDM5A in human cancers, survey the field of KDM5A inhibitors including their anticancer activity and modes of action, and the current challenges and potential opportunities of this field.

## Background

**Fig. 1:**
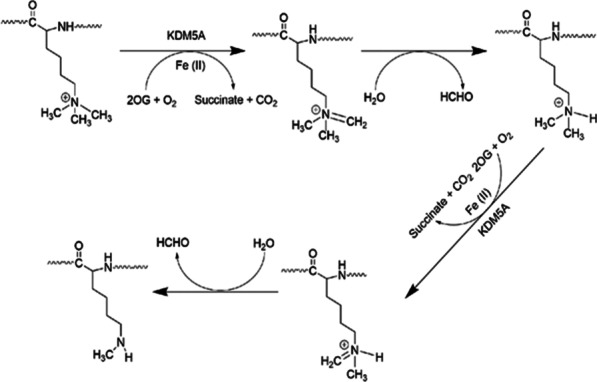
The catalytic mechanism of KDM5A. 2OG: α-ketoglutaric acid

Lysine-specific demethylase 5A (KDM5A), also named Jumonji/ARID domain-containing protein 1A (JARID1A) or retinoblastoma-binding protein 2 (RBP2), originally reported as a retinoblastoma protein (RB) pocket domain-binding protein in 2001 [1], is a Fe(II)- and α-ketoglutaric acid (2OG)-dependent JmjC-containing oxygenase whose demethylase activity was first found in 2007 [2]. KDM5A can eliminate di- and tri-methyl moieties from the fourth lysine of histone 3 (H3K4me2/3), which leads to the activation or repression of transcription [3–13]. Additionally, the fusion gene NUP98-KDM5A, which is produced by rearrangement between NUP98 and KDM5A, mediates hematopoietic cell proliferation and alters myelo-erythropoietic differentiation via demethylating H3K4me2/3 [14–17]. In terms of mechanism, KDM5A and its fusion gene Fe(II)-dependently catalyzes oxidative decarboxylation of 2OG with consumption of O_2_ to generate a reactive iron(IV)-oxo intermediate, carbon dioxide, and succinate. Subsequently, the hemiaminal of the methylated lysine residue fragments to liberate both formaldehyde and an unmethylated lysine residue (Fig. 1) [18–20]. Among the Fe(II)- and 2OG-dependent demethylases, KDM5A exhibits variable levels in the body [21–23]. However, KDM5A showed aberrantly high expression in various solid cancers as well as in acute myeloid leukemia (AML), where it represses differentiation, promotes angiogenesis, drug resistance, and epithelial-mesenchymal transition, enhances adhesion, metastasis, invasiveness, proliferation, and cell motility, and also worsens outcomes [5, 6, 8–10, 14, 21–29]. Therefore, inhibiting KDM5A is potentially an antitumor approach. Herein, we summarize the pivotal roles of KDM5A in cancer progression, advances of research on KDM5A inhibitors and their screening methods, and the prospect of the cancer therapy by KDM5A inhibition.

## Evolutionary and structure of KDM5A

**Fig. 2:**
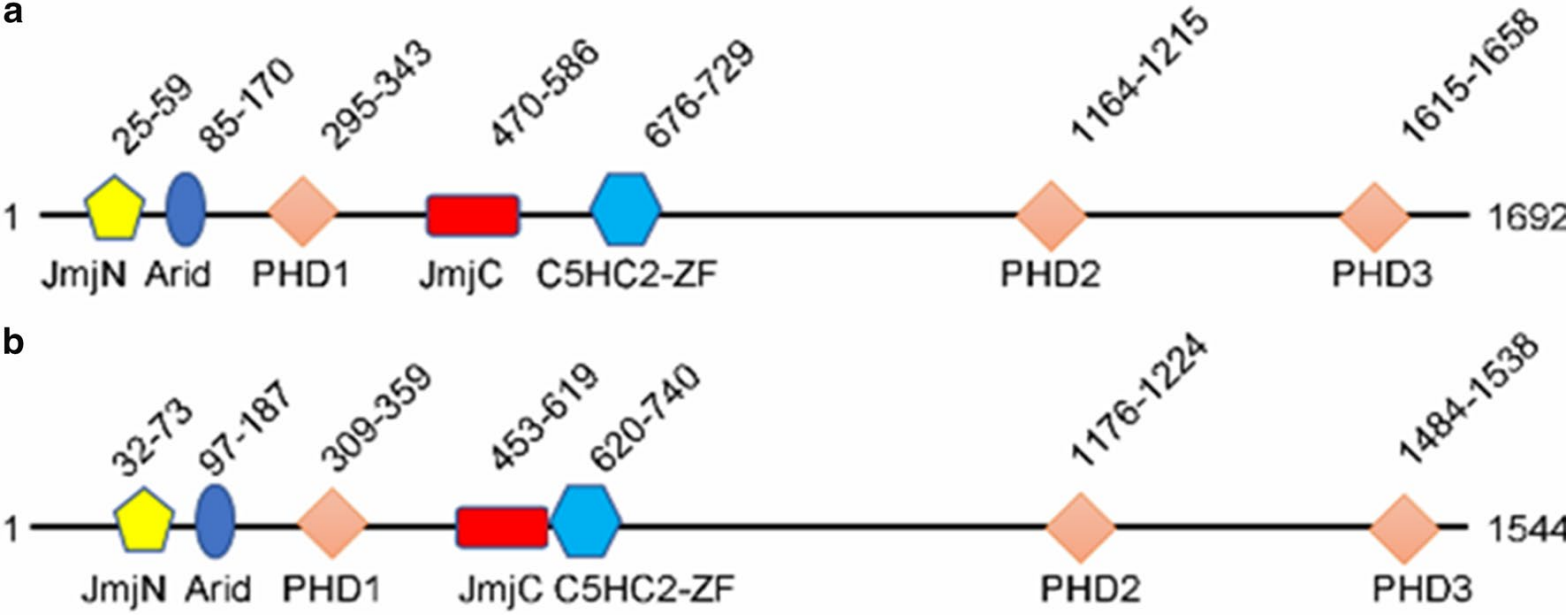
Schematic of the domains of KDM5A and KDM5B

Currently, there are only two types of lysine-specific demethylases (KDMs) found: the flavin-dependent KDM1 family and the 2-oxoglutarate- and oxygen-dependent Jumonji C (JmjC) domain KDMs [20]. The former comprises three members: lysine-specific demethylase 1A (LSD1), LSD1 + 8a, and LSD2 [30–34], while the latter includes 19 members which are dependent on α-ketoglutarate and oxygen to eliminate up to three methyl moieties on lysines [35]. All the members of JmjC KDMs are Fe(II)-dependent oxidation enzymes, because their catalytic amine oxidase domain needs Fe(II). The other domains of these KDMs determine their selectivity for substrates [36]. KDM5A belongs to KDM5 subfamily which comprises four members, KDM5A, KDM5B, KDM5C, and KDM5D. All the KDM5s share several highly conserved that include a Jumonji N (JmjN), a catalytic (JmjC) domain, a helical C5HC2 motif containing zinc finger (C5HC2-ZF) domain, a AT-rich interactive domain (ARID), and two or three plant homeodomain (PHD) domains [37, 38]. KDM5C and KDM5D are located on the X and Y chromosome, respectively [39–45], and they share common domains and biological functions of all KDM5 family members [46]. Meanwhile, KDM5A and KDM5B are located on euchromosomes and have a third PHD domain (PHD3) (Fig. 2) [3, 4, 22, 25, 47–50]. PHDs possess a Cys_4_HisCys_3_ motif that is responsible for coordinating two Zn^2+^ in a cross-brace fashion and recognize histones in a sequence- and modification-dependent manner [51–53]. In demethylases and their assembled complexes, PHDs function as binding units to mediate occupancy and specificity of substrates [54–56]. The PHD1 (KDM5A: residues 295–343, KDM5B: residues 309–359) domain has the strongest binding ability to unmethylated H3K4me and allosterically enhances demethylase activity [38, 57, 58], while it has only 0.2-fold affinity for H3K4me1 than that of unmethylated H3K4 [37]. The function of PHD2 (KDM5A: residues 1164–1215, KDM5B: residues 1176–1224) is unknown. The PHD3 (KDM5A: residues 295–343, KDM5B: residues 309–359) preferably binds to H3K4me3 but also recognizes the other H3K4 methylation states [25, 59]. Out of the PHDs of KDM5A-NUP98, PHD3 specifically binds to H3K4me3 [60]. The KDM5 ARID domain has been shown to recognize specific DNA sequences. Interestingly, although both of KDM5A and KDM5B have an ARID domain (KDM5A: residues 85–170, KDM5B: residues 97–187), the binding consensus has been documented to CCGCCC for KDM5A [61] and GCACA/C for KDM5B [62]. The JmjN (KDM5A: residues 25–59, KDM5B: residues 32–73), originally assumed to always co-occur with catalytic domain JmjC (KDM5A: residues 470–586, KDM5B: residues 453–689) [63], is important for their protein stability [64]. The C5HC2-ZF (KDM5A: residues 679–729, KDM5B: residues 620–740), positioned between the JmjC and PHD2 domains, is a zinc finger with eight potential zinc ligand binding residues and may act as a DNA binding domain [64].

## Roles of KDM5A in homeostasis and disease

KDM5A mediates a range of physiological and pathological events, such as cell motility, stemness, and epithelial-mesenchymal transition (EMT), via activating or repressing transcription in demethylase-dependent or independent manners in both homeostasis and disease [11, 65, 66].

## The roles of KDM5A in homeostasis

**Fig. 3:**
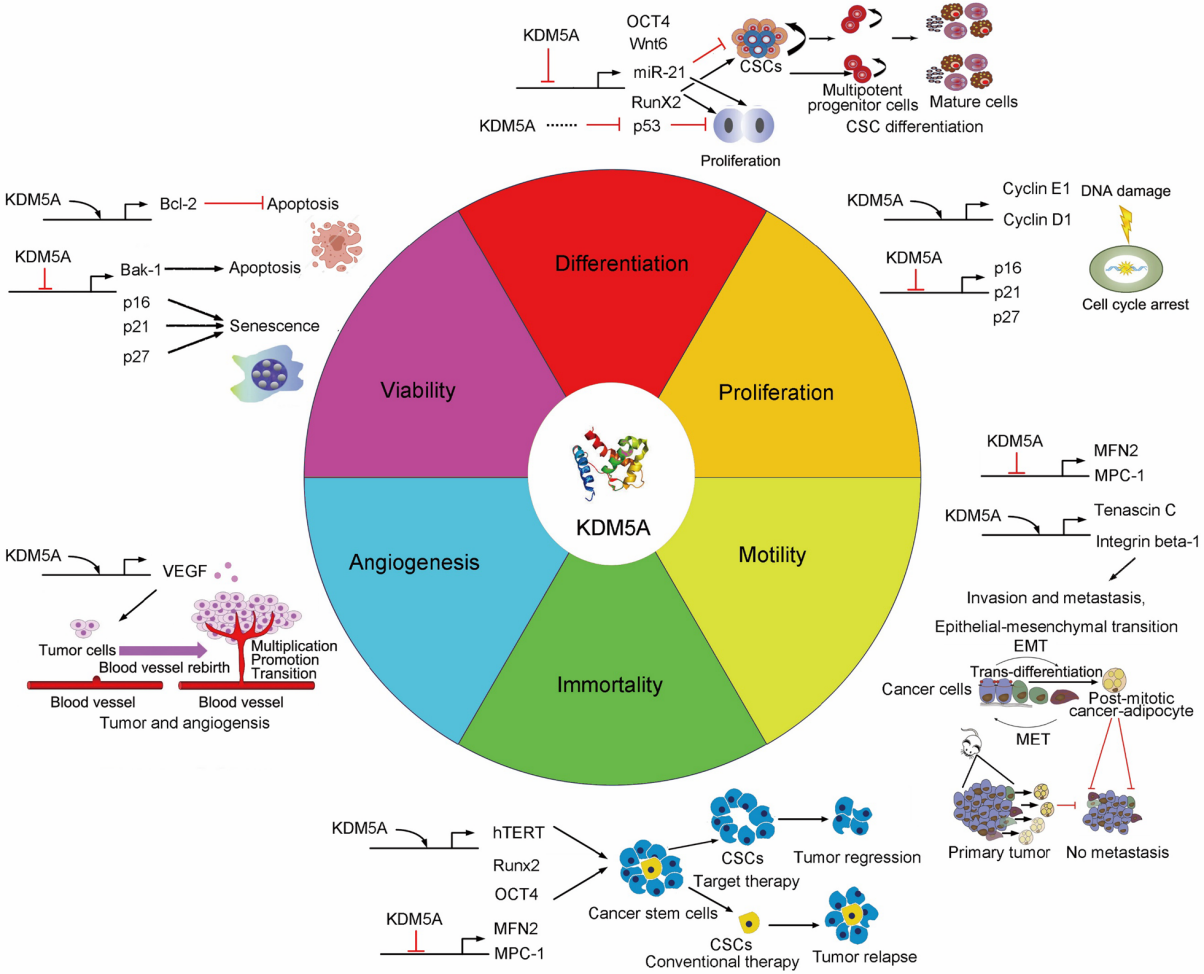
The biological function and action mechanism of KDM5A

KDM5A transcriptionally regulates cell development and differentiation (Table 1) [2, 65, 67–74]. As a general transcriptional corepressor, KDM5A can be transactivated by C/EBPβ and enhances preadipocyte differentiation via blocking Wnt/β-catenin activity in a demethylase-dependent fashion and binding to the *Wnt6* gene promoter and repressing its transcription [75]. KDM5A is also documented to suppress the odontogenic differentiation potentiality of human dental pulp cells by removing H3K4me3 from specific gene promoters [76]. It impedes the reprogramming efficiency of human induced pluripotent stem cells via demethylase-dependently inhibiting *OCT4* transcription (Fig. 3) [71]. In addition, KDM5A is also involved in many other cell events such as cell cycle progression, cellular senescence, circadian rhythm, natural killer cell activation, and social behavior [69, 77–81].

**Table 1:** The roles of KDM5A in homeostasis

Model (cells/tissues/species)	Mechanism	Functions	References
Preadipocytes	C/EBPβ blocks Wnt/β-catenin activity in a demethylase-dependent fashion and represses *Wnt6* transcription	Promoting preadipocyte differentiation	[75]
Reprogramming-resistant fibroblast	KDM5A transcriptionally inhibits *OCT4* expression	Inhibiting reprogramming efficiency	[71]
Mouse embryonic stem cells	KDM5A transcriptionally inhibits cell cycle genes	Repressing cell differentiation	[74–76]
Heart	KDM5A interacts with CLOCK-BMAL1 to bind to the *Per2* promoter, increasing histone acetylation and enhancing transcription in a demethylase-independent fashion	Activating CLOCK-BMAL1 and affecting the circadian clock	[77]
Natural killer cells	KDM5A mediates NK cell activation through interacting with p50 to inhibit *SOCS1*	Activating NK cells	[79]
IMR-90 cells	KDM5A contributes to retinoblastoma-mediated gene silencing	Mediating cellular senescence	[78]
Drosophila	KDM5 regulates immune control and gut microbiota maintenance	Regulating social behavior	[81]

## The roles of KDM5A in non-cancer disease

KDM5A is associated with many non-cancer diseases, such as congenital heart disease (CHD) [82] and bacterial, viral, or parasitic infection [79, 83, 84]. It also mediates renal failure in lipopolysaccharide-induced sepsis of mice [85]. Upregulation of KDM5A inhibits the commitment of marrow-derived mesenchymal stem cells lineage into osteoblasts by decreasing H3K4me3 levels in the promoter region of Runt-related transcription factor 2 (*Runx2*) (Fig. 3)[86].

## KDM5A in human cancer

KDM5A is associated with cancer growth, differentiation, multi-drug resistance, invasion, and metastasis in various cancers (Table 2 and Fig. 3).

**Table 2:** The functional roles of KDM5A in cancer

Cancer type	Mechanism	Functions	References
Acute myeloid leukemia	KDM5A forms fused gene with NUP98 and maintains expression of the *Hoxa* gene cluster via specifically binding their promoters	Blocking genetic program required for normal cellular differentiation	[5, 51, 90, 91]
Breast cancer	KDM5A transcriptionally inhibits expression of *p16, p27,* and *Bak-1,* and induces* ITGB1* expression	Promoting cancer proliferation, drug tolerance, and metastasis	[5, 9, 12, 23, 29, 92–94]
Prostate cancer	KDM5A decrease the levels of two tumor suppression and differentiation genes *KLF4* and *E-cadherin*	Leading to malignancy of PCs	[95–98]
Glioblastoma	KDM5A promotes cell proliferation, self-renewal, and drug resistance of GBM by regulating *Hoxa-9* and -*10* and transcription factor *FOXM*; it promotes migration and invasion of glioma cells via downregulating *ZEB1*	Promoting proliferation, self-renewal, and drug resistance of GBM and inhibiting the migration and invasion of glioma cells	[4, 99, 100]
Lung cancer	KDM5A inhibiting the expression of *p27* and *TFPI2*, and upregulating *cyclin D1*, and *ITGB1*; it promotes angiogenesis and EMT via Akt signaling	Facilitating proliferation, motility, migration, invasion, metastasis, and drug resistance of lung cancer	[13, 25, 101, 102]
Gastric cancer	KDM5A represses its target genes CDKIs (*p16*, *p21,* and *p27*); it transactivates *VEGF* and promotes gastric tumorigenesis	Promoting proliferation metastasis, and angiogenesis	[7, 8, 103, 104]
Hepatocellular carcinoma	KDM5A is negatively regulated by miR-21, and repressed cyclin-dependent kinase inhibitors (CDKIs)	Promoting proliferation and inducing senescence	[105, 106]
Renal cell carcinoma	KDM5A induces stem-like cancer cells and promote RCC in demethylase-dependent manner	Facilitating proliferation, metastasis and inducing stemness of cancer cells	[47, 107]
Pancreatic cancer	KDM5A transcriptionally inhibits *IGF2BP2* expression and *MPC-1*	Promoting aerobic glycolysis and cell proliferation	[108, 109]
Melanoma	KDM5A work as a tumor suppressor gene	Improving the response of melanoma to PD-L1 antibody	[110–112]
Ovarian cancer	KDM5A promotes cancer progression in demethylase-dependent manner	Promoting proliferation EMT, and drug resistance	[6, 113]

### Acute myeloid leukemia (AML)

AML is a cancer with a high lethality that occurs more frequently in older populations [87]. Meanwhile, acute megakaryoblastic leukemia (AMKL), a subtype of AML with similar cell morphology to abnormal megakaryoblasts, accounts for a significant fraction of pediatric AML cases [16, 88]. KDM5A is highly expressed in pediatric AMKL with a cytogenetically cryptic fusion NUP98/NSD1 (t(5; 11)(q35; p15)) [89]. The fusion gene NUP98/KDM5A is formed by the fusion of the C-terminal PHD finger of KDM5A to NUP98 [51]. This fusion is required for leukemogenic transformation, by increasing progenitor cell self-renewal and blocking myeloblast differentiation [51, 90]. The transplantation of bone marrow cells transduced with NUP98-KDM5A into mice resulted in the development of AML. Mechanistically, marked overexpression of Hoxa cluster genes, most notably *Hoxa-5, -7, -9*, and *-10*, is linked with leukemic characteristics [89]. NUP98/JARID1A is involved with promoting expression of the Hoxa gene cluster via specifically binding to their promoters, deregulating the “reader” of histone marks, resulting in the inhibition of the epigenetic program necessary for regular cell differentiation [14]. Furthermore, KDM5A mediates drug resistance to Wee1 inhibition in acute leukemia [91].

### Breast cancer

Breast cancer is the one of the leading causes of female cancer mortality [22]. KDM5A is frequently overexpressed in primary breast cancer cases and increase the proliferation, metastasis, and drug resistance of breast cancer [5, 9, 12, 23, 29, 92–94]. KDM5A is responsible for the proliferation and drug tolerance of HER2-positive breast cancer [5]. KDM5A promotes drug resistance of clinical drugs such as trastuzumab and erlotinib via deregulating *p21* and BCL2-antagonist/killer 1 (*Bak1*) [5, 9, 22, 23], and facilitates the proliferation of many HER2-positive breast cancer cell lines through mediating cell cycle and apoptosis [5, 9, 12, 23, 29, 92]. In triple-negative breast cancer (TNBC), inhibition of KDM5A resulted in anti-cancer activity via impairing cell cycle and senescence by regulating *p16* and *p27* [12, 29]. Apart from demethylase-dependent activity, KDM5A is also involved in metastasis of TNBC via inducing the expression of integrin β-1 (ITGB1) [92].

### Prostate cancer (PCa)

KDM5A is upregulated in PCa tissue compared to normal prostate tissue [95]. KDM5A is also critical for the generation of drug tolerant PCa cells during chronic drug exposure [96]. In addition, KDM5A mediates reduction in methylated H3K4 and thus decreases the levels of two tumor suppression and differentiation genes *KLF4* and *E-cadherin*, which lead to the malignancy of PCa [97]. KDM5A is also capable of promoting PCa progression via the KDM5A/miRNA-495/YTHDF2/m6A-MOB3B axis [98].

### Glioblastoma (GBM)

GBM is a highly lethal cancer due to its ability to infiltrate healthy brain tissue [99]. KDM5A is overexpressed in GBM compared to normal brain tissue and plays multifaced roles in GBM progression depending on the type of GBM [100]. It promotes cell proliferation, self-renewal, and drug resistance of GBM by regulating *Hoxa-9* and *-10* and the transcription factor *FOXM1* in the temozolomide-resistant cell line A172 [99, 100]. It is also documented to inhibit migration and invasion of glioma cells via downregulating *ZEB1* in A172 and LN-229 cells [4].

### Lung cancer

KDM5A is overexpressed in lung cancer tissues and facilitates cell proliferation, invasion, and metastasis of lung cancer via inhibiting the expression of *p27* and upregulating *cyclin D1*, and *ITGB1* [10, 13, 25, 101, 102]. KDM5A directly binds to the promoters of these three genes and transcriptionally modulated their transcripts [10]. In gefitinib-tolerant human small-cell lung cancer PC9 cells, KDM5A specifically inhibits the proliferation drug-tolerant cells without affecting their parent cells via suppressing the expression of tissue factor pathway inhibitor 2 (*TFPI2*) [101]. In non-small cell lung cancer cells, KDM5A promotes HIF-1α-VEGF-induced angiogenesis through Akt and induces the epithelial-mesenchymal transition via down-regulating E-cadherin expression and up-regulating N-cadherin and snail expression through activating Akt [25, 102]. KDM5A also promotes tumorigenesis of small cell lung cancer suppressing target genes *NOTCH1* and *NOTCH2* [13].

### Gastric cancer

KDM5A is overexpressed in gastric cancer and increases cell proliferation and metastasis via repressing cyclin-dependent kinase inhibitors (CDKIs: *p16*, *p21*, and *p27*) [7, 8, 103, 104]. It also facilitates gastric cancer malignancy the through TGF-β1-(p-Smad3)-RBP2-E-cadherin-Smad3 feedback circuit [7]. The CagA-PI3K/AKT-Sp1-RBP2-Cyclin D1 axis may also act as a possible mechanism for gastric epithelial cell malignant transformation [103]. The enhancement of gastric tumorigenesis by KDM5A was linked with transactivation of vascular endothelial growth factor (*VEGF*) expression and increased angiogenesis, suggesting that KDM5A overexpression and *VEGF* activation might play key functions in the progression of human gastric cancer [104].

### Hepatocellular carcinoma (HCC)

KDM5A is a prognostic factor for disease-free survival and overall survival of HCC patients [105]. In HCC, KDM5A is negatively regulated by miR-221, and abrogating KDM5A significantly lowered cell proliferation and induced senescence of HCC cells via significantly upregulated CDKIs [106].

### Renal cell carcinoma

Renal cell carcinoma (RCC) is a leading cause of death among urological cancers. KDM5A facilitates cell proliferation and metastasis via reducing methylated H3K4 [107]. Silencing KDM5A leads to the induction of apoptosis and cell cycle arrest [107]. KDM5A is also a prognostic indicator for RCC, and it promotes EMT to induce stemness in tumor cells [47].

### Pancreatic cancer

In sporadic pancreatic neuroendocrine tumors, KDM5A regulated the tumorigenesis via *insulin-like growth factor 2* (*IGF2BP2*) [108, 109]. Ablation of KDM5A could partially reverse the decreased expression of *IGF2BP2* in *Men1*-deficient pancreatic islet cells [108].

### Melanoma

In melanoma, enhancing KDM5A activity improves the response of melanoma to immune checkpoint blockade programmed cell death protein 1 antibody [110]. In addition, retinoblastoma-binding protein 2-homolog 1 (RBP2-H1), a homolog protein with 54% identity with RBP2 (KDM5A), is downregulated in malignant melanoma [111, 112]. RBP2-H1 directly interacts with pRb and regulates the cell cycle and transcriptional. Loss or down-regulation of RBP2-H1 leads to uncontrolled growth and transformed malignant melanomas.

### Ovarian cancer

KDM5A is also involved the progression of ovarian cancer. It promotes the proliferation and EMT of ovarian cancer and is correlated with paclitaxel resistance in SKOV3 cells [6]. MiR-421 suppresses proliferation, migration, and invasion of SKOV-3 and OVCAR-8 cells via targeting KDM5A [113].

## Therapeutic targeting of KDM5A

### Pharmacological Targeting of KDM5A in Cancer Therapy

KDM5A is a 2OG- and Fe^2+^ -dependent demethylase [37, 38]. Due to the conserved binding sites for to 2OG and Fe^2+^ in JmjC demethylases [1], several 2OG competitive inhibitors and Fe^2+^ chelators have exhibited inhibitory activity against KDM5A [1]. However, despite their potent anti-cancer activity *in cellulo* and in vivo, none of them have been approved in preclinical or clinical trials due to poor selectivity and organ toxicity [24]. The following sections discuss the main categories of KDM5A inhibitors and their pharmaceutical properties.

#### 2OG analogs

**Fig. 4:**
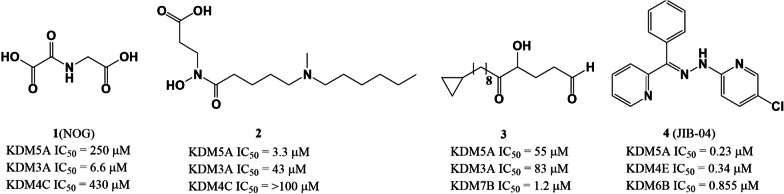
The chemical structures of 2OG analogs

N-Oxalylglycine (NOG, **1**, Fig. 4) is a pan-inhibitor of 2OG oxygenase that acts through binding to the 2OG site and chelation of Fe^2+^ with its C-1 carboxylate and amido groups [114–116]. NOG inhibits KDM5A inhibitor with an IC_50_ value of 250 μM in vitro [5]. Compounds **2**–**4**, containing a 2OG substrate-mimicking hydroxamic acid moiety, are also broad-spectrum JmjC-KDM inhibitors with strong in vitro activity. However, their poor selectivity and low *in cellulo* potency limits their further applications.

#### Isonicotinic acids

**Fig. 5:**
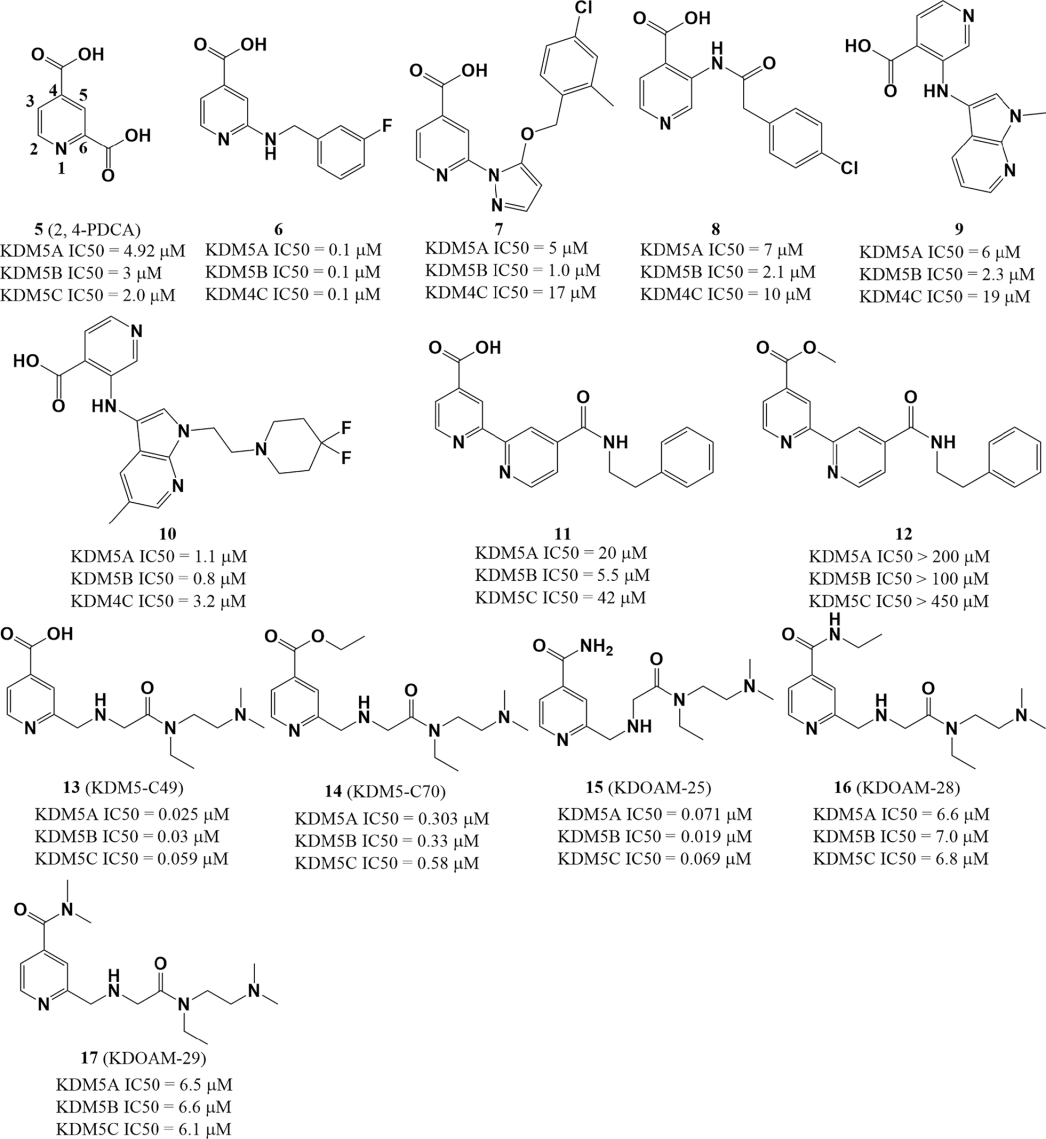
The chemical structures of isonicotinic acids

2,4-Pyridinedicarboxylic acid (2,4-PDCA, **5**) is a pan-demethylase inhibitor with an IC_50_ value of 4.92 µM against KDM5A as measured using an in vitro amplified luminescence proximity homogeneous assay (AlphaScreen assay) [117]. However, **5** also exhibits poor permeability and low selectivity for KDM5A over other KDM5 demethylases [118]. Many analogues of **5** have been synthesized and their structure–activity relationships have been explored to improve the permeability and KDM5A selectivity of **5**. Replacement of the hydrogen at the C5 position of **5** with different groups had a slight effect on KDM5A inhibitory activity (comparing IC_50_ values among compounds **5**–**9**, Fig. 5) [119]. Meanwhile, C6 substitutions **5** would greatly alter inhibitory activity against KDM5A (for example, comparing IC_50_ values for compounds **9**–**11** and **13**, Fig. 5). Moreover, modification of the C4 side group with ethyl or methyl would greatly affect the activity of compounds (comparing IC_50_ values between **11** and **12**; between **13** and **14**; and among **15**, **16**, and **17**, Fig. 5). These results indicated that having a carboxyl group at the C4 position is important for potency against KDM5A [118]. Apart from in vitro activity, the *in cellulo* activity of these isonicotinic acid derivatives were also studied. KDM5-C70 (**14**), an ethyl ester derivative of the most potent hit compound KDM5-C49 (**13**), is cell-permeable but also retains significant in vitro pan-KDM5 inhibitory activity. **14** showed antiproliferative activity against myeloma cells, with genome-wide increase in H3K4me3 observed [114, 115]. KDOAM-25 (**15**), the corresponding amide analogue of **14**, is an efficient and selective histone lysine demethylase 5 (KDM5) antagonist with IC_50_ values < 72 nM for KDM5A-D [116]. This compound globally raises H3K4 methylation at transcription start sites and reduces the proliferation of multiple myeloma (MM1S) cell proliferation.

#### Pyrimidinones

**Fig. 6:**
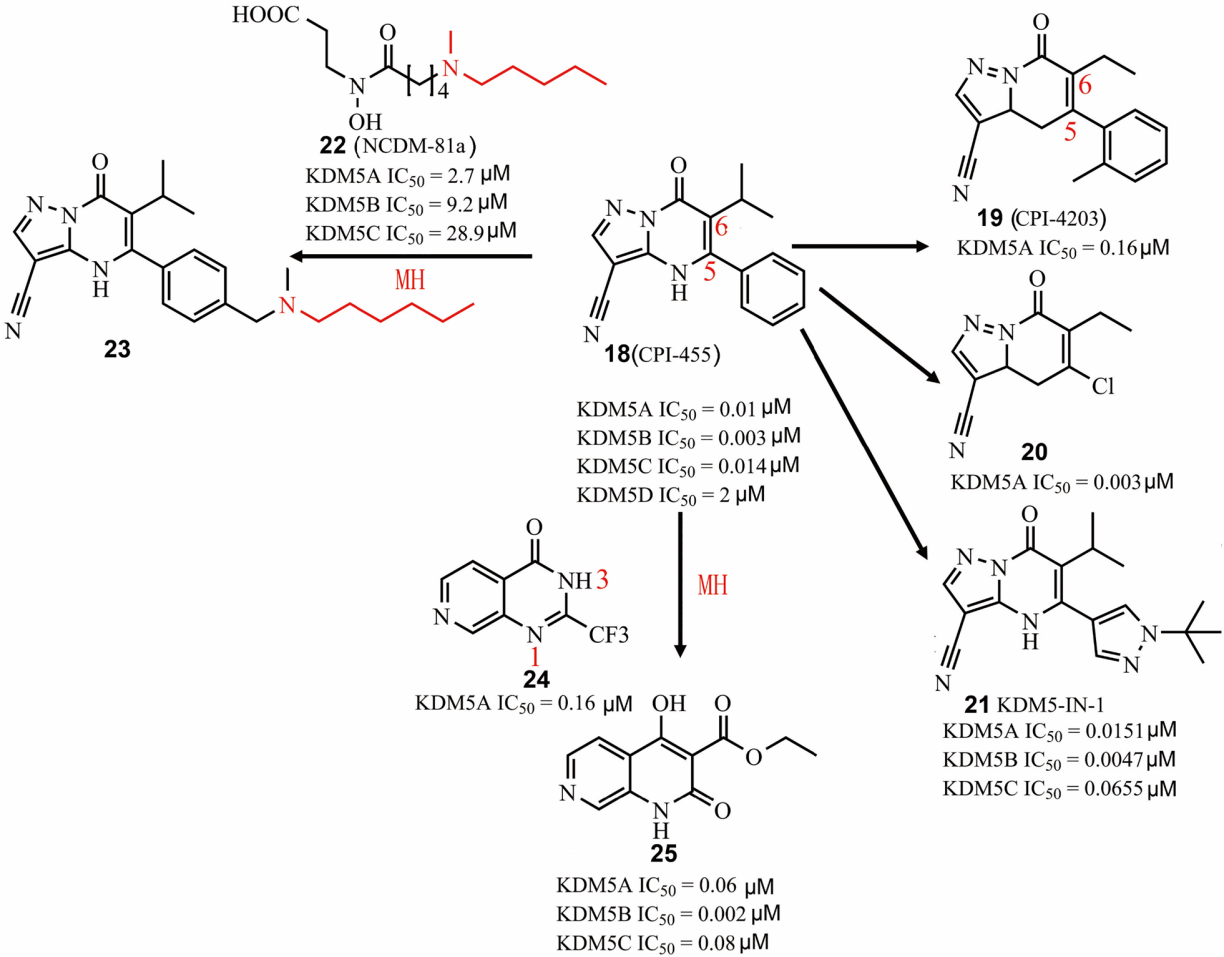
The chemical structures of pyrimidinones. *MH* molecular hybridization

Pyrimidine derivatives were also identified as KDM5A inhibitors in vitro and *in cellulo*. CPI-455 (Fig. 6, **18**) is a selective and pan-KDM5 inhibitor with 10 nM potency against KDM5A [120]. In Hela cells, **18** globally increased H3K4me3 levels [120]. Like other demethylase inhibitors, **18** could also antagonize KDM5B activity (IC_50_ = 3 nM). In accordance with the roles of KDM5A and KDM5B in drug resistance, **18** reduced drug-tolerance by up to 20-fold in several cancer cell models [26]. In addition, **18** showed relatively long retention time (total C_max_ = 192 μM) and excellent oral activity (100 mg/kg) in vivo [26]. CPI-4203 (**19**) is a compound structurally related to **18** but is a less potent KDM5A inhibitor in vitro [26]. Substitution of the C5 position of the o-xylene group of **19** with a chlorine group improved cell potency and pharmacokinetic profile of compound **20** [121]. An optimized compound (**21**) based on comprehensive structure–activity relationship analysis on the C5- and C6-positions of **18** (Fig. 6) showed slightly lower KDM5A inhibition activity (IC_50_ = 15.1 nM) but improved potency against PC9 cells (EC_50_ from 5.2 μM to 0.34 μM) compared to the parent compound [121]. To develop a more potent KDM5A inhibitor *in cellulo*, a hybrid compound (**23**) was made by merging of two fragments NCDM-81a (**22**) (KDM5A IC_50_ = 2.7 μM) and **18**, which showed highly potent and 2-OG competitive inhibition of KDM5A demethylase activity (KDM5A IC_50_ = 0.00437 μM). Moreover, compound **23** could upregulate H3K4Me levels and exhibited highly potent anti-proliferative activity against A549 cells (GI_50_ = 29.6 μM) compared to **18**. Another hybrid compound (**25**) (KDM5B IC_50_ = 0.06 μM) was generated based on molecular hybridization of **24** (KDM5A IC_50_ = 0.16 μM) and **18**. Compound **25** also functioned as a 2-OG competitive KDM5 inhibitor with selectivity over KDM2B and KDM4C. Although **25** exhibited potent activity against KDM5 subfamilies in vitro, it almost has no *in cellulo* inhibitory activity even at 30 μM.

#### Pyrazoles

**Fig. 7:**
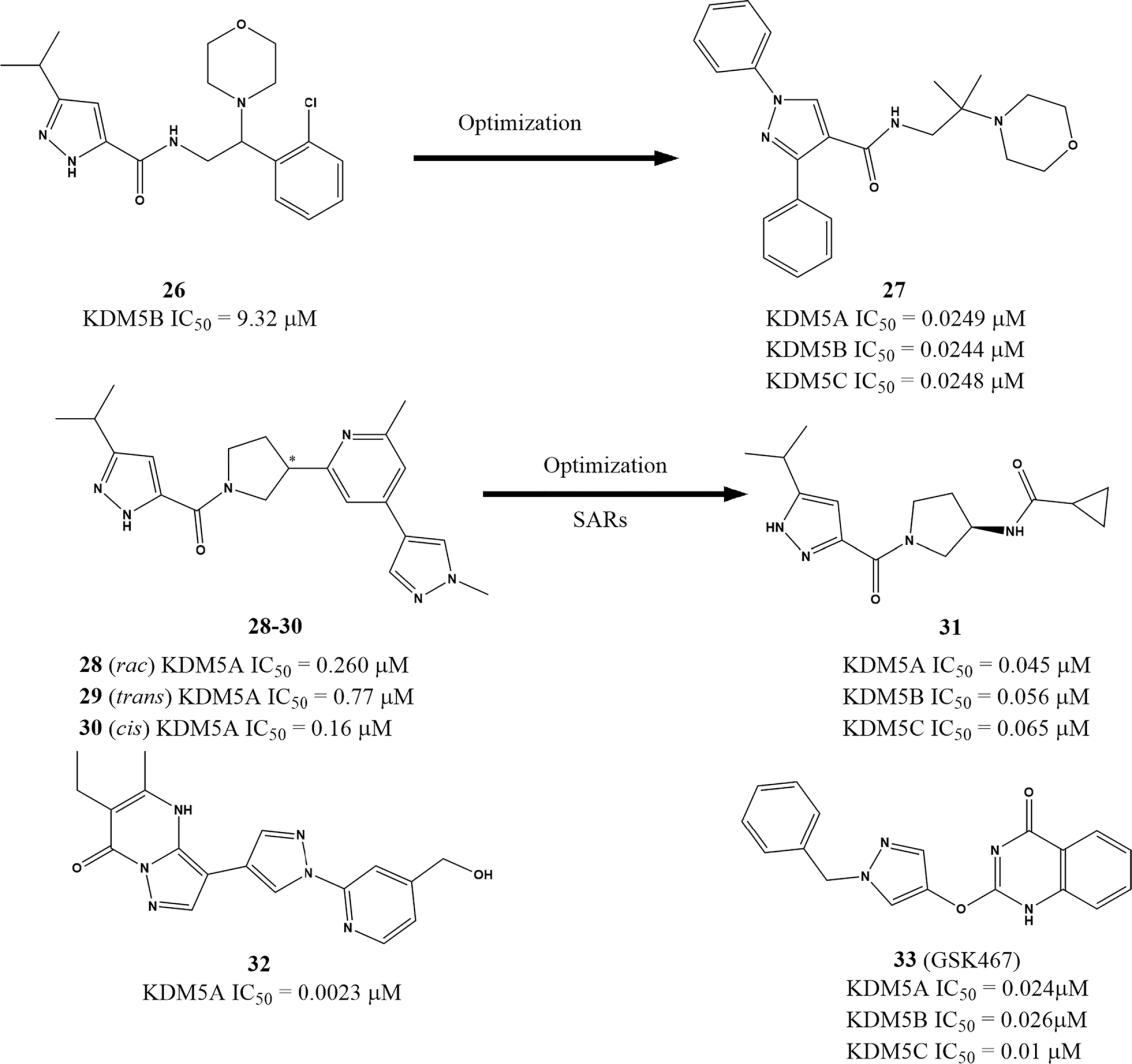
The chemical structures of pyrazoles. *SAR* structure–activity relationship

Liu et al. recently described a pyrazole derivative KDM5B inhibitor **26** (IC_50_ = 9.320 μM) based on structure-based virtual screening from an Enamine library of containing over 20,000,000 molecules. Subsequent optimization led to the generation of 1-(4-methoxyphenyl)-N-(2-methyl-2-morpholinopropyl)-3-phenyl-1H-pyrazole-4-carboxamide (**27**, Fig. 7), a potent and highly selective KDM5 antagonist with IC_50_ values of approx. 25 nM against KDM5A-C. In MKN45 cells, **27** engaged KDM5A and induced accumulation of its substrates H3K4me2 and H3K4Me3, without affecting the levels of H3K4me1, H3K27me2, and H3K9me2/3. A further study also found that **27** has good *in cellulo* activity, as indicated by its ability to inhibit MKN45 cell proliferation, migration and wound healing and [122]. Liang’s group also reported another pyrazole derivative, the racemic **28,** with an IC_50_ value of 0.260 μM (Fig. 7) from high-throughput screening. The *cis*-isomer (**30**, IC_50_ = 0.16 μM) of **28** has about fivefold lower IC_50_ than the *trans*- isomer (**29**, IC_50_ = 0.77 μM). However, even the more potent **26** was ineffective against PC9 cells (EC_50_ > 30 μM) [123]. After structure–activity relationship (SAR) analysis and optimization, a more active compound (**31**) was identified with *in cellulo* (EC_50_ = 960 μM) and in vivo activity. Additionally, **31** showed improved potency, reduced lipophilicity (Log D = 1.3 at pH 7.4), and lower molecular weight compared to the lead compound **30**. Two compounds (**32** and **33**) containing one or two pyrazole rings wares documented and patented as highly potent KDM5A inhibitors with activity in the nanomolar range [124].

#### Heterocyclic compounds

**Fig. 8:**
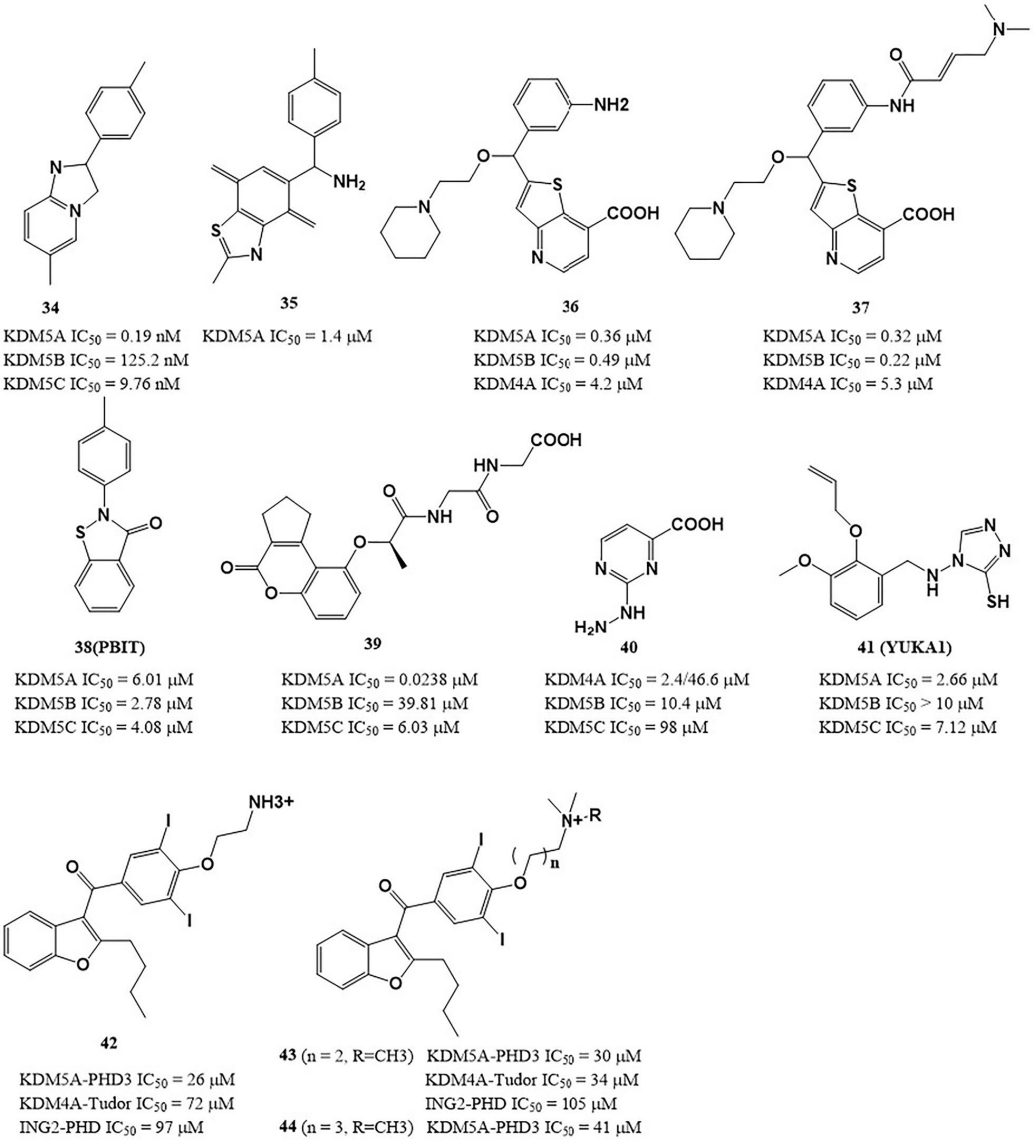
The chemical structures of heterocyclic compounds

Some heterocyclic compounds exhibit good inhibitory activity against KDM5A. Imidazopyridine O4I3 (Fig. 8, **34**) promoted the reprogramming efficiency of pioneering human induced pluripotent stem cell via inhibiting KDM5A and thus enriching H3K4me3 at the *OCT4* promoter [71]. Ryuvidine (Fig. 8, **35**), a KDM5A inhibitor identified by the AlphaScreen method, could prevent generation, and inhibit the growth of gefitinib-tolerant human small-cell lung cancer PC9 cells without affecting parental cells [101]. Compound **37** is an irreversible KDM5A inhibitor, derived from **36** (Fig. 8). **37** contains a (dimethylamine but-2-enamido) phenyl moiety and covalently interacts with Cys481 of KDM5A [125]. PBIT (**38**) inhibits KDM5A with an IC_50_ of 6.01 μM in vitro. Treatment of cancer cells with **38** inhibited removal of H3K4me3, and the inhibition of proliferation was especially effective against cells expressing higher levels of KDM5A [126]. Compound **39** is a potent and specific KDM5A antagonist reported by our group. It displayed significantly greater specificity for KDM5A over KDM4A and other KDM5 family members, and is much more specific than currently described KDM5A antagonists. In the mechanism, **39** blocked the interaction between KDM5A and its substrate H3K4me3, and thus transcriptionally upregulated the levels of *p16* and *p27* levels via increasing occupations of H3K4me3 on their promoters [12]. Tetrazoylhydrazide (Fig. 8, **40**) was initially identified as a KDM4A inhibitor, but it also inhibits KDM5A demethylase activity with an IC_50_ value of 10.4 μM [127]. **40** competitively binds to KDM5A, using its tetrazole moiety as an isostere of the C5-carboxyl group of 2OG. Replacement of the terminal hydrazide of **40** almost abrogated its inhibitory activity against KDM5A, while the extension of two or three carbon atoms for the alkyl chain led to less than twofold loss in potency. Based on high-throughput screening, Gale et al*.* screened and identified a 3-thio-1,2,4-triazole derivative YUKA1 (Fig. 8, **41**) as a KDM5A inhibitor with an in vitro IC_50_ value of 2.66 ± 0.69 µM, this compound reduced gefitinib- and trastuzumab-induced drug tolerance in EGFR-mutant lung cancer cells and HER2 + breast cancer cells, respectively [92]. Aminodarone derivatives (**42**–**44**) are a new class of KDM5A inhibitors with potencies in the ~ 40 μM range, which were identified using a HaloTag assay for displacement of histone H3K4me3 from PHD3 [128]. This study demonstrates the feasibility of targeting noncatalytic domains of JmjC-KDMs for the first time and opens a window for the development of allosteric demethylase inhibitors.

#### Metal complexes

**Fig. 9:**
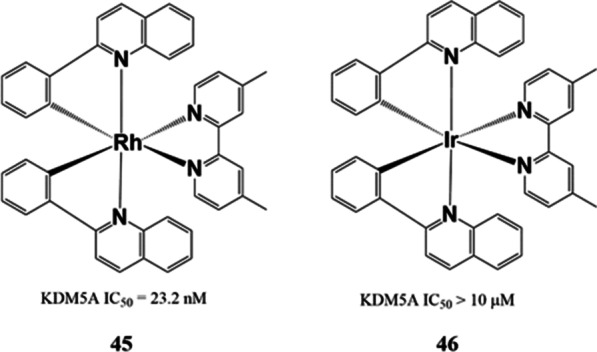
The chemical structures of metal complexes

The rhodium (III) complex **45** (Fig. 9) is the first metal-based antagonist of KDM5A described [29]. It blocked the KDM5A-H3K4me3 interaction in human TNBC cells and increased the amplification of *p27* gene promoters. With an IC_50_ of 23.2 ± 1.8 nM for KDM5A, this complex was selective for KDM5A over other KDMs and additionally exhibited anti-proliferative effects towards TNBC in vivo. Interestingly, **45** exhibited more potent inhibitory activity on KDM5A in vitro and *in cellulo* than its congener **46**.

##### The binding modes of KDM5A inhibitors

Comprehensive efforts have been directed towards characterizing the binding modes of KDM5A interactions with diverse inhibitors. Cheng and Yan’s group have explored the crystal structures of the JmjC domain of KDM5A in complex with the co-factor α-ketoglutarate (αKG), Mn^2+^, and eight KDM5 inhibitors [129]. They determined the complex structure of KDM5A-αKG-Mn^2 +^ and found that metal ion can binds to six ligands (side chains of His-483, Glu-485, and His-571, two oxygen atoms of αKG, and a water molecule) in an octahedral coordination sphere. In the catalytic mechanism, the final site would be bound by an O_2_ molecule to begin the demethylation process via accepting a hydrogen atom from the substrate. The inhibitor **13** binds to KDM5A JmjC domain in a similar fashion to αKG, with RMS deviation of only 0.3 A˚ across 293 pairs of α-C atoms between the two KDM5A structures. The isonicotinic acid group of **13** engages the αKG binding region, with its terminal carboxyl group interacting via H-bonds with Lys-501, Tyr-409 and water, in a similar manner to αKG. Like αKG, **13** provides two ligands for metal coordination, through its pyridine and aminomethyl nitrogen atoms and generates comparable non-polar interactions with KDM5A. However, rather than engaging the 4th and 5th ligand sites as αKG does, **13** moves to sites 5 and 6, while a water molecule switches from the 6th to the 4th site. The distinct binding interactions between αKG and **13** in the bidentate interaction with the metal species results in a conformational shift of the side chain of Asn-493. In the αKG-bound conformation, Asn-493 forms a bridge between αKG and Gln-557, creating a water-free interface between αKG and Asn-493/Gln-557. However, in the **13**-bound structure, Asn-493 H-bonds with a water molecule in the 4th ligand position, while a second water molecule engages with the terminal carboxylate of the isonicotinic acid group. Superimposition of the two structures indicates that Asn-493 shifts via a 180-degree rotation of the side-chain torsional angle c1, producing an interface between **13** and Asn-493/Gln-557 that contains at least three well-structured water molecules. Apart from Asn-493, there is no significant deviation in the active site between the two complexes. All other seven compounds exhibited nearly the same interaction, underscoring the role of the Asn-493 local substructure in mediating catalysis, and also providing a molecular justification for the finding that alteration of the carboxylate (**6–11** and **13**) reduces inhibitory activity significantly. Inhibitor-specific interactions with R-73, Q-85, D-412, W-470, Q-535, N-575, or N-585 were recorded. This binding analysis could be employed for the future development of efficient KDM5A ligands in several ways. Firstly, the chemical space near Cys-481, which is a residue unique to the KDM5A, could be exploited. Secondly, functional groups could be added to protrude into an adjacent water-occupied groove containing multiple unique residues of KDM5A. Third, multiple branches could be combined into a single molecule. Finally, functional groups could be modified to optimize interactions with the contacted residues.

## Conclusion remarks and perspectives

### Perspective and predicament for study KDM5A roles in cancers

Mounting evidence supports that KDM5A is crucial in regulating embryonic stem cell differentiation via subtly controlling gene activation and suppression [130, 131]. KDM5A has variable expression statuses in different cancer tissues and its overexpression is closely related to multi-drug resistance of many cancer cells [59]. Since KDM5A can act as either as a tumor suppressor [4] or an oncogene in cancer [6, 8], its functional profile in different cancer types should be mapped. KDM5A acts by demethylase-dependently regulating mRNA and miRNA transcripts and participates in cancer proliferation, metastasis, and tumorigenesis [4–6, 132]. However, the underlying mechanisms of KDM5A in cancer stemness, drug resistance and autophagy still require further research. There are already some studies that validate the non-demethylase roles of the lysine-specific demethylases (KDMs), which means these enzymes are not just merely methylation erasers. All KDMs possess binding domains that lack catalytic activity, and their associations with chromatin or nucleosomes could be considered as a type of protein–protein or protein-nucleic acid interactions where the covalent methylation/demethylation events play a part. Thus, targeting the KDM binding domains offers potential for novel therapeutic strategies [133]. However, such an approach requires the premise of establishing a method which can selectively detect KDM activity in cells. KDM5A has been documented to promote *tenascin C* (TNC) expression and invasion in a demethylase-independent manner [94]. KDM5A is involved in lengthening of *DICER1* via maintenance of 3′ UTR length [115]. KDM5A also exhibits its demethylase-independent functions via formatting complexes with other proteins. For example, it promotes *Per2* promoter transcription in a demethylase-independent manner through forming complex with the circadian rhythm-regulatory transcription factors CLOCK and BMAL1 [77]. KDM5A also interacts physically with HDAC complexes and reduce radiosensitivity of the HeLa and MCF-7 [134, 135]. All the studies showed that blocking the interaction between KDM5A and its ligands proteins may be an alternative strategy for modulating the non-demethylase activity of KDM5A.

### Perspectives for the future development of KDM5A inhibitors

Several KDM5A inhibitors have displayed promising results against cancers [26, 29], driving further research into developing cell-permeable, potent, and selective KDM5A antagonists. Only one KDM5 inhibitor, GS-5801 developed by Gilead Sciences, has entered phase 1 clinical trials for chronic Hepatitis B in New Zealand and the USA [136]. Therefore, more potent and selective KDM5A inhibitors are urgently needed to help us expound the role for KDM5A in physiological and disease processes and to reduce off-target effects resulting from lack of selectivity. There are several challenges that have to be overcome during development of KDM5A inhibitors. Firstly, the high similarity in the catalytic domain between KDM5A with other demethylases, especially the other members of the KDM5 family, which greatly increases the difficult in developing selective inhibitors. Secondly, KDM5A contributes to the heterogeneity of varieties of cancers via regulating transcriptional outputs in cell or cancer-dependent manner, which is one of the main reasons for KDM5A inhibitors failing in *in cellulo* and in vivo studies. Thus, the identification of the disease profiles of current KDM5A inhibitors is very important for advancing clinical applications. Thirdly, KDM5A inhibitors are also faced with the common challenges during drug design or optimization such as improving cell permeability and stability. Finally, only inhibiting KDM5A demethylase activity may be insufficient to decrease the expression of their target genes.

To address the above challenges, some solutions have already been proposed. Firstly, some non-catalytic domains have been documented to regulate KDM5A demethylase activity and blocking these less highly conserved domains can potentially abrogate the catalytic activity of KDM5A in a selective fashion. Secondly, given the enzymatic properties of KDM5A demethylase, the identification of an allosteric regulatory site and development of allosteric inhibitors is also an alternative strategy for targeting this enzyme. Thirdly, impeding the interaction between KDM5A and its partner proteins may be also a viable strategy to improve the selectivity of KDM5A inhibitors. Fourthly, to improve cell permeability and stability of KDM5A inhibitors, medicinal chemistry principles can be applied to optimize the properties of the lead compounds. Fifthly, plant-derived natural products (PDNPs) have been considered as a unique source of biologically active molecules and scaffolds for drug-discovery [137]. Xue-fu-Zhu-Yu decoction, a famous Chinese herbal formula, has been documented to protect rats from retinal ischemia via inhibiting KDM5A and PKM2 and thus downregulating levels of HIF-1α and VEGF [137]. Results like these suggest that PDNPs may emerging trend for identifying KDM5A inhibitors for multiple cancer therapy. Finally, combination therapy is also a promising avenue to develop KDM5A inhibitors, which may accelerate inhibitors into clinic development. For example, H3K4me3 tends to be highly enriched at active promoter regions adjacent to transcriptional initiation sites. Meanwhile, histone deacetylase 1 (HDAC1) is reported to negatively regulate mitotic chromatin binding of the notch effector RBP-J in a KDM5A-dependent fashion via coregulating their downstream genes. Thus it may be feasible for an HDAC1 inhibitor to be applied synergistically with KDM5A inhibitors to weaken the viability of cancer cells [138]. Other modulators of such as EZH2, E2F4, and small ubiquitin-like modifier 2 may also be feasible for combination with KDM5A inhibitors for cancer therapy [70, 80, 139].

In summary, KDM5A plays important roles in mediating differentiation and gene transcription processes. KDM5A demethylase represents a unique anti-cancer target because its inhibition can be effective at impeding tumor progression and multi-drug resistance in KDM5A-amplified cancers. Continued identification of specific inhibitors of KDM5A is needed to enhance our understanding of this enzyme in cancer biology. However, although preclinical studies of KDM5A inhibitors for cancer therapy have been reported, no specific KDM5A inhibitor has been advanced into clinical trials. Potent and selective KDM5A inhibitors with drug-like characteristics could potentially be used alone or in combination with other differentiation therapies [140], immunotherapeutics [141], or chromatin-targeting agents [142], hold promise for future anticancer therapy.

## Data Availability

Not applicable.

## Abbreviations

KDM5A: Lysine specific demethylase 5A
H3K4me2/3: Di- and tri-methyl groups from the fourth positions on histone 3 protein
CHD: Congenital heart disease
Runx2: Runt-related transcription factor 2
AML: Acute myeloid leukemia
AMKL: Acute megakaryoblastic leukemia
PHD: Plant homeodomain
TNBC: Triple-negative breast cancer
PCa: Prostate cancer
ITGB1: Integrin β-1
TFPI2: Tissue factor pathway inhibitor 2
HCC: Hepatocellular carcinoma
RCC: Renal cell carcinoma
IGF2BP2: Insulin-like growth factor 2
RBP2-H1: Retinoblastoma-binding protein 2-homolog 1
2OG: 2-Oxoglutarate
NOG: N-Oxalylglycine
TNC: Tenascin C
KDMs: Lysine-specific demethylases
SAR: Structure–activity relationship
HDAC1: Histone deacetylase 1

## References

[1] Chan SW, Hong W (2001). Retinoblastoma-binding protein 2 (Rbp2) potentiates nuclear hormone receptor-mediated transcription. J Biol Chem.

[2] Christensen J, Agger K, Cloos PA, Pasini D, Rose S, Sennels L (2007). RBP2 belongs to a family of demethylases, specific for tri-and dimethylated lysine 4 on histone 3. Cell.

[3] Cui J, Quan M, Xie D, Gao Y, Guha S, Fallon MB (2019). A novel KDM5A/MPC-1 signaling pathway promotes pancreatic cancer progression via redirecting mitochondrial pyruvate metabolism. Oncogene.

[4] Dai B, Huang H, Guan F, Zhu G, Xiao Z, Mao B (2018). Histone demethylase KDM5A inhibits glioma cells migration and invasion by down regulating ZEB1. Biomed Pharmacother.

[5] Choi HJ, Joo HS, Won HY, Min KW, Kim HY, Son T (2018). Role of RBP2-induced ER and IGF1R-ErbB signaling in tamoxifen resistance in breast cancer. J Natl Cancer Inst.

[6] Feng T, Wang Y, Lang Y, Zhang Y (2017). KDM5A promotes proliferation and EMT in ovarian cancer and closely correlates with PTX resistance. Mol Med Rep.

[7] Liang X, Zeng J, Wang L, Shen L, Ma X, Li S (2015). Histone demethylase RBP2 promotes malignant progression of gastric cancer through TGF-β1-(p-Smad3)-RBP2-E-cadherin-Smad3 feedback circuit. Oncotarget.

[8] Zeng J, Ge Z, Wang L, Li Q, Wang N, Björkholm M (2010). The histone demethylase RBP2 Is overexpressed in gastric cancer and its inhibition triggers senescence of cancer cells. Gastroenterology.

[9] Hou J, Wu J, Dombkowski A, Zhang K, Holowatyj A, Boerner JL (2012). Genomic amplification and a role in drug-resistance for the KDM5A histone demethylase in breast cancer. Am J Transl Res.

[10] Teng YC, Lee CF, Li YS, Chen YR, Hsiao PW, Chan MY (2013). Histone demethylase RBP2 promotes lung tumorigenesis and cancer metastasis. Cancer Res.

[11] Kirtana R, Manna S, Patra SK (2020). Molecular mechanisms of KDM5A in cellular functions: Facets during development and disease. Exp Cell Res.

[12] Yang GJ, Ko CN, Zhong HJ, Leung CH, Ma DL (2019). Structure-based discovery of a selective KDM5A inhibitor that exhibits anti-cancer activity via inducing cell cycle arrest and senescence in breast cancer cell lines. Cancers.

[13] Oser MG, Sabet AH, Gao W, Chakraborty AA, Schinzel AC, Jennings RB (2019). The KDM5A/RBP2 histone demethylase represses NOTCH signaling to sustain neuroendocrine differentiation and promote small cell lung cancer tumorigenesis. Genes Dev.

[14] Cardin S, Bilodeau M, Roussy M, Aubert L, Milan T, Jouan L (2019). Human models of NUP98-KDM5A megakaryocytic leukemia in mice contribute to uncovering new biomarkers and therapeutic vulnerabilities. Blood Adv.

[15] Hara Y, Shiba N, Yamato G, Ohki K, Tabuchi K, Sotomatsu M (2019). Patients aged less than 3 years with acute myeloid leukaemia characterize a molecularly and clinically distinct subgroup. Br J Haematol.

[16] Hara Y, Shiba N, Ohki K, Tabuchi K, Yamato G, Park MJ (2017). Prognostic impact of specific molecular profiles in pediatric acute megakaryoblastic leukemia in non-Down syndrome. Genes Chromosomes Cancer.

[17] De Rooij JD, Branstetter C, Ma J, Li Y, Walsh MP, Cheng J (2017). Pediatric non-down syndrome acute megakaryoblastic leukemia is characterized by distinct genomic subsets with varying outcomes. Nat Genet.

[18] Zhang J, Jing L, Li M, He L, Guo Z (2019). Regulation of histone arginine methylation/demethylation by methylase and demethylase. Mol Med Rep.

[19] Walport LJ, Hopkinson RJ, Schofield CJ (2012). Mechanisms of human histone and nucleic acid demethylases. Curr Opin Chem Biol.

[20] Walport LJ, Hopkinson RJ, Chowdhury R, Schiller R, Ge W, Kawamura A (2016). Arginine demethylation is catalysed by a subset of JmjC histone lysine demethylases. Nat Commun.

[21] Horton JR, Engstrom A, Zoeller EL, Liu X, Shanks JR, Zhang X (2016). Characterization of a linked jumonji domain of the KDM5/JARID1 family of histone H3 lysine 4 demethylases. J Biol Chem.

[22] Blair LP, Cao J, Zou MR, Sayegh J, Yan Q (2011). Epigenetic regulation by lysine demethylase 5 (KDM5) enzymes in cancer. Cancers.

[23] Paolicchi E, Crea F, Farrar WL, Green JE, Danesi R (2013). Histone lysine demethylases in breast cancer. Crit Rev Oncol Hematol.

[24] Rasmussen PB, Staller P (2014). The KDM5 family of histone demethylases as targets in oncology drug discovery. Epigenomics.

[25] Wang S, Wang Y, Wu H, Hu L (2013). RBP2 induces epithelial-mesenchymal transition in non-small cell lung cancer. PLoS ONE.

[26] Vinogradova M, Gehling VS, Gustafson A, Arora S, Tindell CA, Wilson C (2016). An inhibitor of KDM5 demethylases reduces survival of drug-tolerant cancer cells. Nat Chem Biol.

[27] Paroni G, Bolis M, Zanetti A, Ubezio P, Helin K, Staller P (2019). HER2-positive breast-cancer cell lines are sensitive to KDM5 inhibition: definition of a gene-expression model for the selection of sensitive cases. Oncogene.

[28] Hinohara K, Wu HJ, Vigneau S, McDonald TO, Igarashi KJ, Yamamoto KN (2018). KDM5 histone demethylase activity links cellular transcriptomic heterogeneity to therapeutic resistance. Cancer Cell.

[29] Yang GJ, Wang W, Mok SWF, Wu C, Law BYK, Miao XM (2018). Selective inhibition of lysine-specific demethylase 5A (KDM5A) using a rhodium(III) complex for triple-negative breast cancer therapy. Angew Chem Int Ed Engl.

[30] Gao S, Chen S, Han D, Wang Z, Li M, Han W (2020). Chromatin binding of FOXA1 is promoted by LSD1-mediated demethylation in prostate cancer. Nat Genet.

[31] Coleman DJ, Sampson DA, Sehrawat A, Kumaraswamy A, Sun D, Wang Y (2020). Alternative splicing of LSD1+8a in neuroendocrine prostate cancer is mediated by SRRM4nd metastasis. Neoplasia.

[32] Magliulo D, Bernardi R, Messina S (2018). Lysine-specific demethylase 1A as a promising target in acute myeloid leukemia. Front Oncol.

[33] Chen F, Yang H, Dong Z, Fang J, Wang P, Zhu T (2013). Structural insight into substrate recognition by histone demethylase LSD2/KDM1b. Cell Res.

[34] Zhang Q, Qi S, Xu M, Yu L, Tao Y, Deng Z (2013). Structure-function analysis reveals a novel mechanism for regulation of histone demethylase LSD2/AOF1/KDM1b. Cell Res.

[35] Janardhan A, Kathera C, Darsi A, Ali W, He L, Yang Y (2018). Prominent role of histone lysine demethylases in cancer epigenetics and therapy. Oncotarget.

[36] Tian X, Fang J (2007). Current perspectives on histone demethylases. Acta Biochim Biophys Sin (Shanghai).

[37] Dorosz J, Kristensen LH, Aduri NG, Mirza O, Lousen R, Bucciarelli S (2019). Molecular architecture of the Jumonji C family histone demethylase KDM5B. Sci Rep.

[38] Torres IO, Kuchenbecker KM, Nnadi CI, Fletterick RJ, Kelly MJ, Fujimori DG (2015). Histone demethylase KDM5A is regulated by its reader domain through a positive-feedback mechanism. Nat Commun.

[39] Jangravi Z, Tabar MS, Mirzaei M, Parsamatin P, Vakilian H, Alikhani M (2015). Two splice variants of y chromosome-located lysine-specific Demethylase 5D have distinct function in prostate cancer cell line (DU-145). J Proteome Res.

[40] Jensen LR, Amende M, Gurok U, Moser B, Gimmel V, Tzschach A (2005). Mutations in the JARID1C gene, which is involved in transcriptional regulation and chromatin remodeling, cause X-linked mental retardation. Am J Hum Genet.

[41] Komura K, Jeong SH, Hinohara K, Qu F, Wang X, Hiraki M (2016). Resistance to docetaxel in prostate cancer is associated with androgen receptor activation and loss of KDM5D expression. Proc Natl Acad Sci USA.

[42] Li N, Dhar SS, Chen TY, Kan PY, Wei Y, Kim JH (2016). JARID1D is a suppressor and prognostic marker of prostate cancer invasion and metastasis. Cancer Res.

[43] Iwase S, Lan F, Bayliss P, de la Torre-Ubieta L, Huarte M, Qi HH (2007). The X-linked mental retardation gene SMCX/JARID1C defines a family of histone H3 lysine 4 demethylases. Cell.

[44] Link JC, Wiese CB, Chen X, Avetisyan R, Ronquillo E, Ma F (2020). X chromosome dosage of histone demethylase KDM5C determines sex differences in adiposity. J Clin Investig.

[45] Rondinelli B, Schwerer H, Antonini E, Gaviraghi M, Lupi A, Frenquelli M (2015). H3K4me3 demethylation by the histone demethylase KDM5C/JARID1C promotes DNA replication origin firing. Nucleic Acids Res.

[46] Brier A-SB, Loft A, Madsen JG, Rosengren T, Nielsen R, Schmidt SF (2017). The KDM5 family is required for activation of pro-proliferative cell cycle genes during adipocyte differentiation. Nucleic Acids Res.

[47] Zhou D, Kannappan V, Chen X, Li J, Leng X, Zhang J (2016). RBP2 induces stem-like cancer cells by promoting EMT and is a prognostic marker for renal cell carcinoma. Exp Mol Med.

[48] Yamane K, Tateishi K, Klose RJ, Fang J, Fabrizio LA, Erdjument-Bromage H (2007). PLU-1 is an H3K4 demethylase involved in transcriptional repression and breast cancer cell proliferation. Mol Cell.

[49] Barrett A, Madsen B, Copier J, Lu PJ, Cooper L, Scibetta AG (2002). PLU-1 nuclear protein, which is upregulated in breast cancer, shows restricted expression in normal human adult tissues: a new cancer/testis antigen?. Int J Cancer.

[50] Gong F, Clouaire T, Aguirrebengoa M, Legube G, Miller KM (2017). Histone demethylase KDM5A regulates the ZMYND8–NuRD chromatin remodeler to promote DNA repair. J Cell Biol.

[51] Sanchez R, Zhou MM (2011). The PHD finger: a versatile epigenome reader. Trends Biochem Sci.

[52] Musselman CA, Kutateladze TG (2011). Handpicking epigenetic marks with PHD fingers. Nucleic Acids Res.

[53] Taverna SD, Li H, Ruthenburg AJ, Allis CD, Patel DJ (2007). How chromatin-binding modules interpret histone modifications: lessons from professional pocket pickers. Nat Struct Mol Biol.

[54] Horton JR, Upadhyay AK, Qi HH, Zhang X, Shi Y, Cheng X (2010). Enzymatic and structural insights for substrate specificity of a family of jumonji histone lysine demethylases. Nat Struct Mol Biol.

[55] Wen H, Li J, Song T, Lu M, Kan PY, Lee MG (2010). Recognition of histone H3K4 trimethylation by the plant homeodomain of PHF2 modulates histone demethylation. J Biol Chem.

[56] Lan F, Collins RE, De Cegli R, Alpatov R, Horton JR, Shi X (2007). Recognition of unmethylated histone H3 lysine 4 links BHC80 to LSD1-mediated gene repression. Nature.

[57] Longbotham JE, Chio CM, Dharmarajan V, Trnka MJ, Torres IO, Goswami D (2019). Histone H3 binding to the PHD1 domain of histone demethylase KDM5A enables active site remodeling. Nat Commun.

[58] Petronikolou N, Longbotham JE, Fujimori DG (2020). Extended Recognition of the Histone H3 Tail by Histone Demethylase KDM5A. Biochenmistry.

[59] Sharma SV, Lee DY, Li B, Quinlan MP, Takahashi F, Maheswaran S (2010). A chromatin-mediated reversible drug-tolerant state in cancer cell subpopulations. Cell.

[60] Wang GG, Song J, Wang Z, Dormann HL, Casadio F, Li H (2009). Haematopoietic malignancies caused by dysregulation of a chromatin-binding PHD finger. Nature.

[61] Tu S, Teng YC, Yuan C, Wu YT, Chan MY, Cheng AN (2008). The ARID domain of the H3K4 demethylase RBP2 binds to a DNA CCGCCC motif. Nat Struct Mol Biol.

[62] Scibetta AG, Santangelo S, Coleman J, Hall D, Chaplin T, Copier J (2007). Functional analysis of the transcription repressor PLU-1/JARID1B. Mol Cell Biol.

[63] Huang F, Chandrasekharan MB, Chen YC, Bhaskara S, Hiebert SW, Sun ZW (2010). The JmjN domain of Jhd2 is important for its protein stability, and the plant homeodomain (PHD) finger mediates its chromatin association independent of H3K4 methylation. J Biol Chem.

[64] Clissold PM, Ponting CP (2001). JmjC: cupin metalloenzyme-like domains in jumonji, hairless and phospholipase A2β. Trends Biochem Sci.

[65] Klose RJ, Yan Q, Tothova Z, Yamane K, Erdjument-Bromage H, Tempst P (2007). The retinoblastoma binding protein RBP2 is an H3K4 demethylase. Cell.

[66] Plch J, Hrabeta J, Eckschlager T (2019). KDM5 demethylases and their role in cancer cell chemoresistance. Int J Cancer.

[67] Benevolenskaya EV, Murray HL, Branton P, Young RA, Kaelin WG (2005). Binding of pRB to the PHD protein RBP2 promotes cellular differentiation. Mol Cell.

[68] Lopez-Bigas N, Kisiel TA, DeWaal DC, Holmes KB, Volkert TL, Gupta S (2008). Genome-wide analysis of the H3K4 histone demethylase RBP2 reveals a transcriptional program controlling differentiation. Mol Cell.

[69] Beshiri ML, Holmes KB, Richter WF, Hess S, Islam AB, Yan Q (2012). Coordinated repression of cell cycle genes by KDM5A and E2F4 during differentiation. Proc Natl Acad Sci USA.

[70] Pasini D, Hansen KH, Christensen J, Agger K, Cloos PA, Helin K (2008). Coordinated regulation of transcriptional repression by the RBP2 H3K4 demethylase and Polycomb-Repressive Complex 2. Genes Dev.

[71] Dabiri Y, Gama-Brambila RA, Taškova K, Herold K, Reuter S, Adjaye J (2019). Imidazopyridines as potent KDM5 demethylase inhibitors promoting reprogramming efficiency of human iPSCs. iScience.

[72] Kong SY, Kim W, Lee HR, Kim HJ (2017). The histone demethylase KDM5A is required for the repression of astrocytogenesis and regulated by the translational machinery in neural progenitor cells. FASEB J.

[73] Fellous A, Earley RL, Silvestre F (2019). The Kdm/Kmt gene families in the self-fertilizing mangrove rivulus fish, Kryptolebias marmoratus, suggest involvement of histone methylation machinery in development and reproduction. Gene.

[74] Eid W, Abdel-Rehim W (2016). Vitamin C promotes pluripotency of human induced pluripotent stem cells via the histone demethylase JARID1A. Biol Chem.

[75] Guo L, Guo YY, Li BY, Peng WQ, Tang QQ (2019). Histone demethylase KDM5A is transactivated by the transcription factor C/EBPβ and promotes preadipocyte differentiation by inhibiting Wnt/β-catenin signaling. J Biol Chem.

[76] Li QM, Li JL, Feng ZH, Lin HC, Xu Q (2020). Effect of histone demethylase KDM5A on the odontogenic differentiation of human dental pulp cells. Bioengineered.

[77] DiTacchio L, Le HD, Vollmers C, Hatori M, Witcher M, Secombe J (2011). Histone lysine demethylase JARID1a activates CLOCK-BMAL1 and influences the circadian clock. Science.

[78] Chicas A, Kapoor A, Wang X, Aksoy O, Evertts AG, Zhang MQ (2012). H3K4 demethylation by Jarid1a and Jarid1b contributes to retinoblastoma-mediated gene silencing during cellular senescence. Proc Natl Acad Sci USA.

[79] Zhao D, Zhang Q, Liu Y, Li X, Zhao K, Ding Y (2016). H3K4me3 demethylase Kdm5a is required for NK cell activation by associating with p50 to suppress SOCS1. Cell Rep.

[80] Huang C, Cheng J, Bawa-Khalfe T, Yao X, Chin YE, Yeh ET (2016). SUMOylated ORC2 recruits a histone demethylase to regulate centromeric histone modification and genomic stability. Cell Rep.

[81] Chen K, Luan X, Liu Q, Wang J, Chang X, Snijders AM (2019). Drosophila histone demethylase KDM5 regulates social behavior through immune control and gut microbiota maintenance. Cell Host Microbe.

[82] Szot JO, Cuny H, Blue GM, Humphreys DT, Ip E, Harrison K, Szot JO, Cuny H, Blue GM, Humphreys DT, Ip E, Harrison K (2018). A screening approach to identify clinically actionable variants causing congenital heart disease in exome data. Circ Genom Precis Med.

[83] Syn G, Anderson D, Blackwell JM, Jamieson SE (2017). Toxoplasma gondii infection is associated with mitochondrial dysfunction in vitro. Front Cell Infect Microbiol.

[84] Kralickova P, Milota T, Litzman J, Malkusova I, Jilek D, Petanova J (2019). CVID-associated tumors: Czech nationwide study focused on epidemiology, immunology and genetic background in a cohort of patients with CVID. Front Immunol.

[85] Liu Y, Yu Y, Zhang J, Wang C (2019). The therapeutic effect of dexmedetomidine on protection from renal failure via inhibiting KDM5A in lipopolysaccharide-induced sepsis of mice. Life Sci.

[86] Wang C, Wang J, Li J, Hu G, Shan S, Li Q (2016). KDM5A controls bone morphogenic protein 2-induced osteogenic differentiation of bone mesenchymal stem cells during osteoporosis. Cell Death Dis.

[87] Hansrivijit P, Gale RP, Barrett J, Ciurea SO (2019). Cellular therapy for acute myeloid Leukemia-Current status and future prospects. Blood Rev..

[88] Roussy M, Bilodeau M, Jouan L, Tibout P, Laramée L, Lemyre E (2018). NUP98-BPTF gene fusion identified in primary refractory acute megakaryoblastic leukemia of infancy. Genes Chromosomes Cancer..

[89] de Rooij JD, Hollink IH, Arentsen-Peters ST, van Galen JF, Berna Beverloo H, Baruchel A (2013). NUP98/JARID1A is a novel recurrent abnormality in pediatric acute megakaryoblastic leukemia with a distinct HOX gene expression pattern. Leukemia..

[90] Gough SM, Slape CI, Aplan PD (2011). NUP98 gene fusions and hematopoietic malignancies: common themes and new biologic insights. Blood..

[91] Garcia TB, Uluisik RC, van Linden AA, Jones KL, Venkataraman S, Vibhakar R (2020). Increased HDAC Activity and c-MYC Expression Mediate Acquired Resistance to WEE1 Inhibition in Acute Leukemia. Front Oncol..

[92] Gale M, Sayegh J, Cao J, Norcia M, Gareiss P, Hoyer D (2016). Screen-identified selective inhibitor of lysine demethylase 5A blocks cancer cell growth and drug resistance. Oncotarget.

[93] Ham J, Lee S, Lee H, Jeong D, Park S, Kim SJ (2018). Genome-Wide Methylation Analysis Identifies NOX4 and KDM5A as Key Regulators in Inhibiting Breast Cancer Cell Proliferation by Ginsenoside Rg3. Am J Chin Med..

[94] Cao J, Liu Z, Cheung WK, Zhao M, Chen SY, Chan SW (2014). Histone demethylase RBP2 is critical for breast cancer progression and metastasis. Cell Rep..

[95] Vieira FQ, Costa-Pinheiro P, Ramalho-Carvalho J, Pereira A, Menezes FD, Antunes L (2014). Deregulated expression of selected histone methylases and demethylases in prostate carcinoma. Endocr Relat Cancer..

[96] Yan H, Chen X, Zhang Q, Qin J, Li H, Liu C (2011). Drug-tolerant cancer cells show reduced tumor-initiating capacity: depletion of CD44 cells and evidence for epigenetic mechanisms. PloS one..

[97] Huang PH, Chen CH, Chou CC, Sargeant AM, Kulp SK, Teng CM (2011). Histone deacetylase inhibitors stimulate histone H3 lysine 4 methylation in part via transcriptional repression of histone H3 lysine 4 demethylases. Mol Pharmacol..

[98] Du C, Lv C, Feng Y, Yu S (2020). Activation of the KDM5A/miRNA-495/YTHDF2/m6A-MOB3B axis facilitates prostate cancer progression. J Exp Clin Cancer Res..

[99] Banelli B, Carra E, Barbieri F, Würth R, Parodi F, Pattarozzi A (2015). The histone demethylase KDM5A is a key factor for the resistance to temozolomide in glioblastoma. Cell Cycle..

[100] Romani M, Daga A, Forlani A, Pistillo MP, Banelli B (2019). Targeting of histone demethylases KDM5A and KDM6B inhibits the proliferation of temozolomide-resistant glioblastoma cells. Cancers..

[101] Mitsui E, Yoshida S, Shinoda Y, Matsumori Y, Tsujii H, Tsuchida M (2019). Identification of ryuvidine as a KDM5A inhibitor. Sci Rep..

[102] Qi L, Zhu F, Li SH, Si LB, Hu LK, Tian H (2014). Retinoblastoma binding protein 2 (RBP2) promotes HIF-1α-VEGF-induced angiogenesis of non-small cell lung cancer via the Akt pathway. PloS one..

[103] Liang X, Zeng J, Wang L, Shen L, Li S, Ma L (2014). Histone demethylase RBP2 induced by Helicobactor Pylori CagA participates in the malignant transformation of gastric epithelial cells. Oncotarget..

[104] Li L, Wang L, Song P, Geng X, Liang X, Zhou M (2014). Critical role of histone demethylase RBP2 in human gastric cancer angiogenesis. Mol Cancer..

[105] Wang ZY, Yang J, Liu CK, Shen SQ (2017). High Expression of Retinoblastoma-Binding Protein 2 (RBP2) in Patients with Hepatocellular Carcinoma and Its Prognostic Significance. Med Sci Monit..

[106] Liang X, Zeng J, Wang L, Fang M, Wang Q, Zhao M (2013). Histone demethylase retinoblastoma binding protein 2 is overexpressed in hepatocellular carcinoma and negatively regulated by hsa-miR-212. PloS one..

[107] Kumar A, Kumari N, Sharma U, Ram S, Singh SK, Kakkar N (2019). Reduction in H3K4me patterns due to aberrant expression of methyltransferases and demethylases in renal cell carcinoma: prognostic and therapeutic implications. Sci Rep..

[108] Lin W, Watanabe H, Peng S, Francis JM, Kaplan N, Pedamallu CS (2015). Dynamic epigenetic regulation by menin during pancreatic islet tumor formation. Mol Cancer Res..

[109] Huang S, Wu Z, Cheng Y, Wei W, Hao L (2019). Insulin-like growth factor 2 mRNA binding protein 2 promotes aerobic glycolysis and cell proliferation in pancreatic ductal adenocarcinoma via stabilizing GLUT1 mRNA. Acta Biochim Biophys Sin (Shanghai)..

[110] Wang L, Gao Y, Zhang G, Li D, Wang Z, Zhang J (2020). Enhancing KDM5A and TLR activity improves the response to immune checkpoint blockade. Sci Transl Med..

[111] Roesch A, Becker B, Meyer S, Wild P, Hafner C, Landthaler M (2005). Retinoblastoma-binding protein 2-homolog 1: a retinoblastoma-binding protein downregulated in malignant melanomas. Mod Pathol..

[112] Vogt T, Kroiss M, McClelland M, Gruss C, Becker B, Bosserhoff AK (1999). Deficiency of a novel retinoblastoma binding protein 2-homolog is a consistent feature of sporadic human melanoma skin cancer. Lab Invest..

[113] Ren F, Shrestha C, Shi H, Sun F, Zhang M, Cao Y (2020). Targeting of KDM5A by miR-421 in human ovarian cancer suppresses the progression of ovarian cancer cells. Onco Targets Ther..

[114] Johansson C, Velupillai S, Tumber A, Szykowska A, Hookway ES, Nowak RP (2016). Structural analysis of human KDM5B guides histone demethylase inhibitor development. Nat Chem Biol..

[115] Blair LP, Liu Z, Labitigan RL, Wu L, Zheng D, Xia Z (2016). KDM5 lysine demethylases are involved in maintenance of 3'UTR length. Sci Adv..

[116] Tumber A, Nuzzi A, Hookway ES, Hatch SB, Velupillai S, Johansson C (2017). Potent and selective KDM5 inhibitor stops cellular demethylation of H3K4me3 at transcription start sites and proliferation of MM1S myeloma cells. Cell Chem Biol..

[117] Korczynska M, Le D, Younger N, Gregori-Puigjané E, Tumber A, Krojer T (2016). Docking and linking of fragments to discover jumonji histone demethylase inhibitors. J Med Chem..

[118] Rose N, Ng S, Mecinović J, Liénard B, Bello S, Sun Z (2008). Inhibitor scaffolds for 2-oxoglutarate-dependent histone lysine demethylases. J Med Chem..

[119] Jaikhan P, Buranrat B, Itoh Y, Chotitumnavee J, Kurohara T, Suzuki T (2019). Identification of ortho-hydroxy anilide as a novel scaffold for lysine demethylase 5 inhibitors. Bioorg Med Chem Lett..

[120] Gehling VS, Bellon SF, Harmange JC, LeBlanc Y, Poy F, Odate S (2016). Identification of potent, selective KDM5 inhibitors. Bioorg Med Chem Lett..

[121] Liang J, Zhang B, Labadie S, Ortwine DF, Vinogradova M, Kiefer JR (2016). Lead optimization of a pyrazolo[1,5-a] pyrimidin-7(4H)-one scaffold to identify potent, selective and orally bioavailable KDM5 inhibitors suitable for in vivo biological studies. Bioorg Med Chem Lett..

[122] Zhao B, Liang Q, Ren H, Zhang X, Wu Y, Zhang K (2020). Discovery of pyrazole derivatives as cellular active inhibitors of histone lysine specific demethylase 5B (KDM5B/JARID1B). Eur J Med Chem..

[123] Liang J, Labadie S, Zhang B, Ortwine D, Patel S, Vinogradova M (2017). From a novel HTS hit to potent, selective, and orally bioavailable KDM5 inhibitors. Bioorg Med Chem Lett..

[124] Albrecht BK, Bellon SF, Gehling VS, Harmange J-C, LeBlanc Y, Liang J, et al. Therapeutic compounds and uses thereof. U.S. Patent, 2015; Application No. 14/477, 566.

[125] Horton JR, Woodcock CB, Chen Q, Liu X, Zhang X, Shanks J (2018). Structure-based engineering of irreversible inhibitors against histone lysine demethylase KDM5A. J Med Chem..

[126] Itoh Y, Sawada H, Suzuki M, Tojo T, Sasaki R, Hasegawa M (2015). Identification of Jumonji AT-rich interactive domain 1A inhibitors and their effect on cancer cells. ACS Med Chem Lett..

[127] Rüger N, Roatsch M, Emmrich T, Franz H, Schüle R, Jung M (2015). Link A: Tetrazolylhydrazides as selective fragment-like inhibitors of the JumonjiC-domain-containing histone demethylase KDM4A. Chem Med Chem..

[128] Wagner E, Nath N, Flemming R, Feltenberger J, Denu J (2012). Identification and characterization of small molecule inhibitors of a plant homeodomain finger. Biochemistry.

[129] Horton JR, Liu X, Gale M, Wu L, Shanks JR, Zhang X (2016). Structural basis for KDM5A histone lysine demethylase inhibition by diverse compounds. Cell Chem Biol..

[130] Omuro A, DeAngelis LM (2013). Glioblastoma and other malignant gliomas: a clinical review. JAMA..

[131] Wang WP, Tzeng TY, Wang JY, Lee DC, Lin YH, Wu PC (2012). The EP300, KDM5A, KDM6A and KDM6B chromatin regulators cooperate with KLF4 in the transcriptional activation of POU5F1. PloS one.

[132] Zhou M, Zeng J, Wang X, Wang X, Huang T, Fu Y (2015). Histone demethylase RBP2 decreases miR-21 in blast crisis of chronic myeloid leukemia. Oncotarget.

[133] Thinnes CC, England KS, Kawamura A, Chowdhury R, Schofield CJ, Hopkinson RJ (2014). Targeting histone lysine demethylases-progress, challenges, and the future. BBA-Gene Regul Mech..

[134] Varier RA, Carrillo de Santa Pau E, van der Groep P, Lindeboom RG, Matarese F (2016). Recruitment of the mammalian histone-modifying EMSY complex to target genes is regulated by ZNF131. J Biol Chem..

[135] Penterling C, Drexler G, Böhland C, Stamp R, Wilke C, Braselmann H (2016). Depletion of histone demethylase Jarid1A resulting in histone hyperacetylation and radiation sensitivity does not affect dna double-strand break repair. PloS one.

[136] Gilmore S, Tam D, Dick R, Appleby T, Birkus G, Willkom M (2017). Antiviral activity of GS-5801, a liver-targeted prodrug of a lysine demethylase 5 inhibitor, in a hepatitis B virus primary human hepatocyte infection model. J Hepatol..

[137] Tan S, Geng X, Liu J, Pan W, Wang L, Liu H (2017). Xue-fu-Zhu-Yu decoction protects rats against retinal ischemia by downregulation of HIF-1α and VEGF via inhibition of RBP2 and PKM2. BMC Complement Altern Med..

[138] Dreval K, Lake RJ, Fan H-Y (2019). HDAC1 negatively regulates selective mitotic chromatin binding of the Notch effector RBPJ in a KDM5A-dependent manner. Nucleic Acids Res..

[139] Zargar ZU, Kimidi MR, Tyagi S (2017). Dynamic site-specific recruitment of RBP2 by pocket protein p130 modulates H3K4 methylation on E2F-responsive promoters. Nucleic Acids Res..

[140] Schenk T, Chen W, Göllner S, Howell L, Jin L, Hebestreit K (2012). Inhibition of the LSD1 (KDM1A) demethylase reactivates the all-trans-retinoic acid differentiation pathway in acute myeloid leukemia. Nat Med..

[141] Maio M, Covre A, Fratta E, Di Giacomo A, Taverna P, Natali P, Coral S, Sigalotti L (2015). Molecular pathways: at the crossroads of cancer epigenetics and immunotherapy. Clin Cancer Res..

[142] Huang Y, Vasilatos S, Boric L, Shaw P, Davidson N (2012). Inhibitors of histone demethylation and histone deacetylation cooperate in regulating gene expression and inhibiting growth in human breast cancer cells. Breast Cancer Res Treat..

